# Measuring Patient Compliance With Remote Monitoring Following Discharge From Hospital After Major Surgery (DREAMPath): Protocol for a Prospective Observational Study

**DOI:** 10.2196/30638

**Published:** 2022-04-06

**Authors:** Pramit Khetrapal, Ronnie Stafford, Pádraig Ó Scanaill, Huriye Kocadag, Constantinos Timinis, Angela H L Chang, Adamos Hadjivasiliou, Yansong Liu, Olivia Gibbs, Eleanor Pickford, David Walker, Hilary Baker, Jacqueline Duncan, Melanie Tan, Norman Williams, James Catto, Ivana Drobnjak, John Kelly

**Affiliations:** 1 University College London London United Kingdom; 2 University College London Hospital London United Kingdom; 3 University of Sheffield Sheffield United Kingdom

**Keywords:** digital health, telemonitoring, remote monitoring, telehealth, surgery, hospital, compliance, patient monitoring, wearable technology, smart devices

## Abstract

**Background:**

The incidence of major surgery is on the rise globally, and more than 20% of patients are readmitted to hospital following discharge from hospital. During their hospital stay, patients are monitored for early detection of clinical deterioration, which includes regularly measuring physiological parameters such as blood pressure, heart rate, respiratory rate, temperature, and pulse oximetry. This monitoring ceases upon hospital discharge, as patients are deemed clinically stable. Monitoring after discharge is relevant to detect adverse events occurring in the home setting and can be made possible through the development of digital technologies and mobile networks. Smartwatches and other technological devices allow patients to self-measure physiological parameters in the home setting, and Bluetooth connectivity can facilitate the automatic collection and transfer of this data to a secure server with minimal input from the patient.

**Objective:**

This paper presents the protocol for the DREAMPath (Domiciliary Recovery After Medicalization Pathway) study, which aims to measure compliance with a multidevice remote monitoring kit after discharge from hospital following major surgery.

**Methods:**

DREAMPath is a single-center, prospective, observational, cohort study, comprising 30 patients undergoing major intracavity surgery. The primary outcome is to assess patient compliance with wearable and interactive smart technology in the first 30 days following discharge from hospital after major surgery. Secondary outcomes will explore the relation between unplanned health care events and physiological data collected in the study, as well as to explore a similar relationship with daily patient-reported outcome measures (Quality of Recovery–15 score). Secondary outcomes will be analyzed using appropriate regression methods. Cardiopulmonary exercise testing data will also be collected to assess correlations with wearable device data.

**Results:**

Recruitment was halted due to COVID-19 restrictions and will progress once research staff are back from redeployment. We expect that the study will be completed in the first quarter of 2022.

**Conclusions:**

Digital health solutions have been recently made possible due to technological advances, but urgency in rollout has been expedited due to COVID-19. The DREAMPath study will inform readers about the feasibility of remote monitoring for a patient group that is at an increased risk of acute deterioration.

**Trial Registration:**

ISRCTN Registry ISRCTN62293620; https://www.isrctn.com/ISRCTN62293620

**International Registered Report Identifier (IRRID):**

DERR1-10.2196/30638

## Introduction

Patients undergoing major abdominal and pelvic surgery have readmission rates of over 20% in the first 90 days after surgery [1-3]. Furthermore, 23% of postoperative deaths occur after hospital discharge, of which over 95% occur during the first 3 weeks following discharge [4]. Previously described risk factors for readmission to hospital include reduced functional capacity, chronic inflammatory lung disease, and previous anticoagulation therapy [5-7]. However, consensus is lacking, and patients are therefore not routinely monitored after discharge as patients are deemed to be medically fit. The availability of noninvasive home monitoring devices offers the opportunity to collect and report individualized physiological data, which can serve as surrogates for performance status in the home setting. Home monitoring can also include a means to collect patient-reported outcome measures (PROMs) using easy-to-use media. Such a framework would supplement the standard care discharge pathway, which consists of providing patient information on red-flag symptoms and interval outpatient appointments.

Home monitoring of patients with chronic conditions has previously been shown to be effective, for example, in the management of hypertension and congestive heart failure. A study of patients with hypertension found that during a 48-week period, 91.0% of participants measured their blood pressure regularly [8]. A randomized controlled trial comparing the titration of medication based on self-monitoring of blood pressure versus in-clinic blood pressure measurement in patients with hypertension reported that self-monitoring leads to significantly lower blood pressure [9]. Patient compliance with intermittent self-monitoring of weight and vital signs is similarly high, and benefits have been reported for interactions following changes in physiological status such as a reduction in hospital readmissions; this was found to reduce hospital length of stay from 9.5 days to 0.8 days per patient per year [10]. These reports support that patients can engage with home monitoring and that it is feasible. Furthermore, these studies suggest that engaging patients in the home setting with their own health can help inform their medical team of their health status, encourage patient empowerment, and lead to better health outcomes.

In addition to physiological measurements, PROMs, such as the Quality of Recovery (QoR) questionnaire, have been validated for measuring health status in the postoperative period [11]. The QoR tool and others are not used in routine clinical practice but have been successfully used in clinical trials as a measure of recovery and to discriminate between intervention and control arms [12]. This questionnaire can be applied across a wide range of operative procedures, and its 3 versions (QoR-40, QoR-15, and QoR-9) have been validated with domains for physical and mental well-being [13]. The QoR-40 has been used to track patients for up to 1 month following surgery. To date, the tool has not been used in the context of identifying postdischarge decline, although it has been reported that a low QoR score is independently associated with the development of postoperative complications [14]. As smart technology can be used to collect the QoR, the potential exists to utilize the tool in the postdischarge setting to monitor patients.

Smart devices are similar to their traditional electronic counterparts, but with the added feature of connecting to other devices or networks via different wireless protocols such as Bluetooth, near-field communication, Wi-Fi, 3G, etc. Smart technology has been applied to various medical devices suitable for home use. For example, pacemaker checks can now be safely performed remotely, reducing additional hospital visits [15]. Additionally, consumer-grade, wrist-worn pedometers and heart-rate monitors are commonplace, and many commercially available sphygmomanometers, pulse oximeters and other smart devices are able to wirelessly sync newly collected readings to smartphones. Smartphones can in turn upload data, which can facilitate remote monitoring. In theory, a smart health device can transmit data in real time, allowing for immediate identification and early intervention if deemed clinically necessary. Potential benefits of remote monitoring would enable quicker triage of deteriorating patients, to select patients for clinical review, and to further stratify patients requiring readmission to the index hospital of surgery.

In this study, we will explore the use of smart technology using devices to collect postdischarge data similar to the National Early Warning Score and PROMs in patients who were discharged from hospital after major intracavity surgery. The overarching aim of the study is to test the feasibility of using smart technology to collect physiological data and PROMs from this group of patients.

## Methods

### Study Design

This is a prospective, single-center, observational study to assess patient compliance with smart technology devices using the Home and Locally Observed (HALO) kit. The study will recruit patients undergoing any major intracavity surgery, which is associated with a readmission rate of >15% within 30 days of surgery or >20% in the first 90 days of surgery.

### Objectives

#### Primary Objective

The primary objective of the study is to assess patient compliance with wearable and interactive smart technology in the 30 days following discharge from hospital after major intracavity surgery.

#### Secondary Objectives

The secondary objectives are (1) to explore the relation between unplanned health care events and physiological measurements and PROMs in the postdischarge setting for patients undergoing major surgery, and (2) to explore the correlation between PROMs (QoR-15) and physiological measures in patients in the postdischarge setting.

### Recruitment and Participation Criteria

DREAMPath will recruit patients attending anesthesia and surgical preassessment clinics. It is important to keep patient withdrawals from the study to a minimum; however, a patient may withdraw from the study at any time without prejudice to his or her subsequent treatment.

#### Inclusion Criteria

The inclusion criteria are as follows:

- Must be over 18 years of age;
- Scheduled to undergo or has recently undergone major intracavity surgery with a readmission rate of >15% within 30 days or >20% within 90 days;
- Able to provide informed written consent to participate.

#### Exclusion Criteria

The exclusion criteria are as follows:

- Deemed unfit for surgery;
- Unable or unwilling to comply with remote monitoring for any reason;
- Unable or unwilling to fill in a questionnaire in English.

#### Power Calculation

As this is a feasibility study, we aim to recruit 30 patients during this period. It is anticipated that compliance will be achieved for at least 27 of the 30 patients (90%). If 27 patients are observed to comply, then an exact 1-sided 90% CI suggests that the compliance rate will be at least as high as 82%. The timetable for each patient will be 1 calendar month, and patients will be interviewed at the end of the study to collect readmission event data, as well as feedback regarding ease of use.

### Study Period

Patients will provide consent either prior to having surgery or in the immediate postoperative period while they are still in hospital. At the time of consent, patients will be familiarized with the smart devices and mobile app described. If the patients undergo cardiopulmonary exercise testing (CPET) as part of their routine assessment, the results for this will be recorded. This data will be used to compare the performance of CPET data and smart device data in predicting postoperative complications.

The 4-week monitoring period will commence upon discharge from hospital. Patients will be expected to wear the wearable device at all times, engage with other devices (see the *Remote Monitoring Equipment* section below for the list of devices) twice a day, and fill out 1 daily questionnaire. Compliance will be defined as completion of 70% of the daily PROM questionnaires or wearing the wrist-worn trackers for at least 10 hours per day over the study duration. A minimum of 1 reading in each hour of either step count or heart rate will constitute a successful hour of data collection. Patients will be given the opportunity to contact dedicated personnel in the study team for troubleshooting purposes only. Furthermore, the app provides clear feedback to patients to signal the successful collection of study data. We hope that this measure will help patients feel supported to engage with the study and reduce patient withdrawal. If patients are admitted to the hospital during the monitoring period, they will not be expected to bring the HALO kit to the hospital, and the admission period and subsequent use will not factor into their compliance rate.

The end of the study will be defined as completion of a 30-day follow-up after discharge from the hospital. Patients will be seen at the clinic or contacted by phone to conclude their participation. Postdischarge events will be collected at this time, which is defined as any unscheduled contact with a health care professional, which includes, but is not limited to, general practitioner (GP) visits, accident and emergency (A&E) visits, home visits by primary care team, etc.

**Figure 1:**
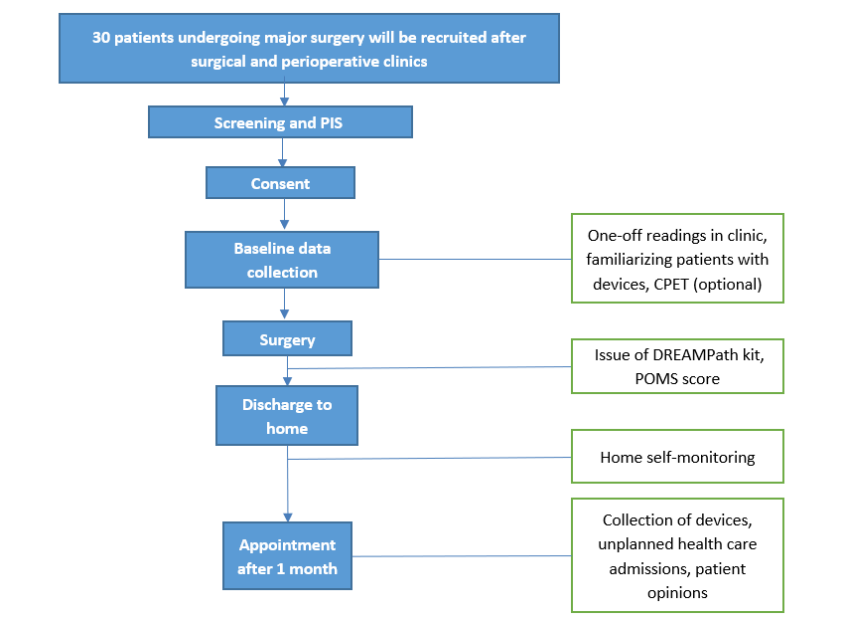
Flow diagram illustrating visits and assessments for patients participating in the DREAMPath (Domiciliary Recovery After Medicalization Pathway) study. CPET: cardiopulmonary exercise testing; PIS: patient information sheet; POMS: postoperative morbidity score.

All study assessments are summarized in Table 1, and the study flowchart is illustrated in Figure 1. As DREAMPath is an observational study, patients will be able to participate in other trials.

**Table 1:** Assessments for patients who consent to participate in the DREAMPath (Domiciliary Recovery After Medicalization Pathway) study.

Visit	Baseline	Postop	First 30 days after discharge	Appointment after 30 days
Identification and enrollment	✓			
Medical history	✓			
Consent and enrollment	✓			
CPET^a^ (optional substudy)^b^	✓			
HALO^c^ data collection (baseline)	✓			
POMS^d^ score (day 5, postoperatively)^b^		✓		
Blood pressure, pulse oximetry, and temperature (twice daily)			✓	✓
Continuous wrist-worn tracker			✓	✓
QoR-15^e^ questionnaire (once daily)	✓		✓	✓
CD^f^ classification at 1-month postop				✓
Adverse events log and patient preferences				✓

^a^CPET: cardiopulmonary exercise testing.

^b^Optional components depending on clinical pathway.

^c^HALO: Home and Locally Observed.

^d^POMS: postoperative morbidity score.

^e^QoR-15: Quality of Recovery–15.

^f^CD: Clavien-Dindo.

### Remote Monitoring Equipment

**Figure 2:**
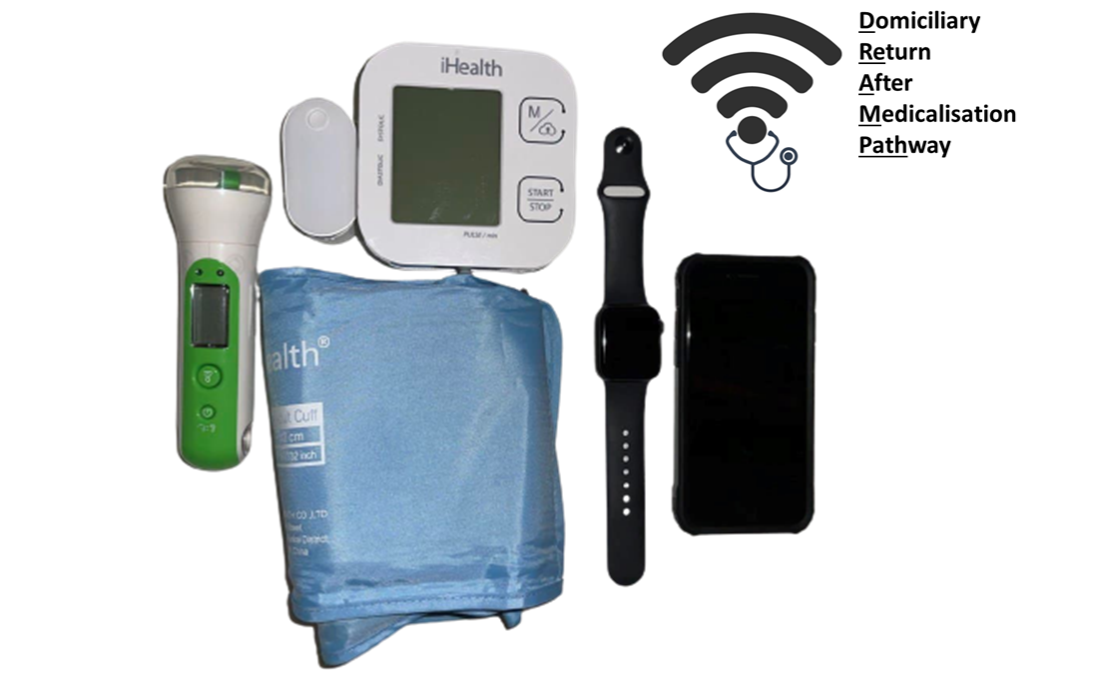
Smart devices issued to patients for twice-daily use: bluetooth enabled thermometer, puse oximeter, sphygmomanometer, Apple Watch and iPhone 7 (from left to right).

The HALO kit is designed to be used with a cellular broadband device, such as a 4G-capable mobile phone, which can be used to complete questionnaires electronically. The broadband device remotely uploads data to an anonymized database. Patients will be issued a HALO kit before discharge from hospital and will be monitored remotely for 30 days. The HALO kit consists of 4 devices: a wrist-worn device with step count and heart rate measurement capability, an iHealth Air pulse oximeter, an iHealth Track blood pressure monitor, and a Koogeek Thermometer. These devices are shown in Figure 2. Patients without a smartphone will be provided with one for the duration of the study. Patients will be instructed to wear the tracker continuously and will be shown how to measure blood pressure, pulse oximetry, and temperature twice daily (morning and evening). All readings will be transmitted from the respective device to the iPhone wirelessly, with the option for patients to self-enter data if they prefer to. Additionally, they will be asked to complete a QoR-15 questionnaire once daily.

Patients will be registered and provided with a trial identifier to ensure all transmitted data are anonymized. They will be contacted via telephone to confirm activation of the system. At the time of consent, patients will be informed that the data collected will not be reviewed for clinical decision-making and that the data are anonymized. Unplanned health care engagement such as GP visits, A&E visits, and readmissions will be collected as “events.” Postoperative complications will be recorded per the Clavien-Dindo classification and the postoperative morbidity score. An exploratory study to understand whether there is a relation between the measured physical parameters and health care events will be conducted. If a derangement of physiological parameters can reliably predict an unplanned health care engagement event, a similar remote monitoring solution could be used to triage patients at home instead of the current standard of care in which patients self-present to primary or emergency care facilities. Upon completion of the study, patients will be asked to return the devices, and information about unplanned health care events and adverse events will be collected. Patients will not be held responsible for any lost or damaged devices. Study devices will not have any patient-identifiable data; and in the event of a lost device, they will be remotely wiped using Apple’s remote wipe feature on iOS. Upon return, devices will be cleaned and sterilized in line with hospital ambulatory device policy.

### Data Collection System

Data is sent over HTTPS (Hypertext Transfer Protocol Secure) from the patients’ phones to our backend server hosted at our affiliated university. The backend consists of a web application framework with a REST application programming interface. Each request delivered from a patient’s mobile phone app is processed in the backend by extracting request payloads and then transforming the data so that they can be stored in a normalized database. All data were pseudo-anonymized, thus ensuring patient privacy. Patients do not have direct access to their data through the app, although interaction with the clinical team means that they can request their own data. A web-based secure portal has also been created so that anonymized data could be analyzed and hypotheses formed for the future creation of analytics and modeling.

### Patient and Public Involvement

This study was planned with patient and public involvement (PPI) at various stages. Prior to planning the study, PPI meetings were held to gather patient views on remote monitoring. In these sessions, patients were asked to share their views on remote monitoring in general, remote monitoring using wearable and smart devices, as well as familiarity with technology-based solutions. Additionally, the study protocol was submitted to The Urology Foundation (TUF) for peer review as part of the TUF Research Scholarship application. Shortlisting is performed on the basis of PPI and scientific feedback, followed by an interview with a panel consisting of PPI and scientific committee members.

### Availability of Data

The study team will control the final anonymized data set. Requests for access will be reviewed by the trial management group, subject to existing contractual arrangements with the sponsor and funders.

### Ethics and Dissemination

The study has ethical approval from the South West – Cornwall & Plymouth Research Ethics Committee (REC reference 18/SW/0206) and has been registered with the ISRCTN Registry (ISRCTN62293620).

The results of the study will be published in peer-reviewed publications and will be presented at relevant national and international conferences. We will work with our patient panel to develop lay reports to disseminate our research findings to patient groups and the clinical teams at participating sites.

## Results

### Status and Timeline

The DREAMPath study is currently underway at our center. Due to the COVID-19 pandemic, research staff were redeployed to provide clinical cover, but we expect to complete recruitment as planned by the end of the first quarter of 2022.

## Discussion

### Benefits of the Study

The main objective of this study is to measure patient compliance with remote monitoring devices using a multidevice kit for patients in the community setting. This is an important step in order to measure patient engagement prior to any large-scale testing of clinical validity of a remote early warning system in a cohort of patients recovering after major surgery. Similar studies have been reported from prior to the smartphone era [16] and have relied on telemetry to collect data with patients actively reporting data. In the study by Kleinpell et al [16], 725 alarms were generated during a 3-month monitoring period of 10 patients, but only 6 of these alarms led to a clinician consult after phone triage. For remote monitoring to be adopted into routine care, false-negatives need to be kept to a minimum but must still capture all major clinical events. During our data analysis of secondary endpoints, we will explore different early warning systems such as modified versions of the national early warning system [17,18] as well as machine-learning–driven algorithms. Due to the large amount of data being produced, a processing pipeline will be required to ensure that clinical resources are not overburdened by the implementation of remote monitoring.

The current study is designed to be an observational study. Data collected in the study will not be accessible to clinicians until after the completion of the monitoring period, as the study ethics do not allow for clinicians to use this data for clinical decision-making. According to the Medicines and Healthcare Products Regulatory Agency (MHRA) guidelines, any remote monitoring medical apps used that influence clinical decision-making are subject to approval [19]. Similar policies exist in other places including the United States, Australia, and the European Union [20]. If our secondary objective analysis shows that it is feasible to use remote monitoring to pre-empt clinical events, an important next step will be to comply with MHRA and other guidance to ensure that this model is validated for use to facilitate clinical adoption. With a marketplace full of mobile apps that can improve health, regulation is extremely important to ensure that end users are not given false assurances about their health. However, the burden of responsibility must not be passed on to patients to interpret medical data without clinical expertise.

### Strengths and Limitations

This is a proof-of-concept remote monitoring study that aims to measure patient compliance with remote monitoring that could potentially improve postoperative care and reduce hospital readmission. The primary outcome will inform us whether remote monitoring is feasible, and the secondary outcomes will inform us of the clinical usefulness of the data collected.

One limitation is that patients will have access to their own data, which may influence their decision to seek medical attention. Further, as this is a pilot study, the sample size is small. A larger study will be necessary to make conclusions about the overall usefulness of remote monitoring.

### Conclusion

Health care services have undergone a digital revolution during the COVID-19 pandemic. Hospitals were routinely giving patients medical devices to self-monitor, and this initiative was supported by national organizations such as NHS England [21]. This is based on evidence that the prehospital measurements of pulse oximetry can be a red flag for patients who may be experiencing “silent hypoxia” due to COVID-19 infection. However, these devices did not have any way of interacting directly with hospital systems and patients were asked to maintain a diary of readings, which can be reviewed by clinicians via remote consultation or A&E attendances. Our study was conceived prior to the pandemic, but offers an important improvement to the current pathway as patients do not need to enter any data manually as the devices included sync data directly using Bluetooth and our bespoke app allows for an automatic upload. After the completion of this study, we hope to integrate our backend server to communicate directly with hospital electronic health record systems to allow seamless data access for clinicians while patients are at home.

## Abbreviations

A&E: accident and emergency
CPET: cardiopulmonary exercise testing
DREAMPath: Domiciliary Recovery After Medicalization Pathway
GP: general practitioner
HALO: Home and Locally Observed
HTTPS: Hypertext Transfer Protocol Secure
MHRA: Medicines and Healthcare Products Regulatory Agency
PPI: patient and public involvement
PROM: patient-reported outcome measure
QoR: Quality of Recovery
TUF: The Urology Foundation
